# Enhanced olfactory sensitivity in autism spectrum conditions

**DOI:** 10.1186/2040-2392-5-53

**Published:** 2014-11-20

**Authors:** Chris Ashwin, Emma Chapman, Jessica Howells, Danielle Rhydderch, Ian Walker, Simon Baron-Cohen

**Affiliations:** Autism Research Centre, Department of Psychiatry, University of Cambridge, Douglas House, 18b Trumpington Road, Cambridge, CB2 8AH UK; Department of Psychology, University of Bath, Bath, BA2 7AY UK; Cambridgeshire and Peterborough NHS Foundation Trust, CLASS Clinic, Cambridge, CB21 5EF UK

**Keywords:** Asperger syndrome, Autism, Autistic traits, Olfaction, Sensory hypersensitivity

## Abstract

**Background:**

People with autism spectrum conditions (ASC) report heightened olfaction. Previous sensory experiments in people with ASC have reported hypersensitivity across visual, tactile, and auditory domains, but not olfaction. The aims of the present study were to investigate olfactory sensitivity in ASC, and to test the association of sensitivity to autistic traits.

**Methods:**

We recruited 17 adult males diagnosed with ASC and 17 typical adult male controls and tested their olfactory sensitivity using the Alcohol Sniff Test (AST), a standardised clinical evaluation of olfactory detection. The AST involves varying the distance between subject and stimulus until an odour is barely detected. Participants with ASC also completed the Autism Spectrum Quotient (AQ) as a measure of autism traits.

**Results:**

The ASC group detected the odour at a mean distance of 24.1 cm (SD =11.5) from the nose, compared to the control group, who detected it at a significantly shorter mean distance of 14.4 cm (SD =5.9). Detection distance was independent of age and IQ for both groups, but showed a significant positive correlation with autistic traits in the ASC group (r =0.522).

**Conclusions:**

This is the first experimental demonstration, as far as the authors are aware, of superior olfactory perception in ASC and showing that greater olfactory sensitivity is correlated with a higher number of autistic traits. This is consistent with results from previous findings showing hypersensitivity in other sensory domains and may help explain anecdotal and questionnaire accounts of heightened olfactory sensitivity in ASC. Results are discussed in terms of possible underlying neurophysiology.

## Background

High-functioning autism (HFA) and Asperger syndrome (AS) are autism spectrum conditions (ASC; also referred to as ASD) diagnosed based on difficulties in social interaction and communication alongside unusually narrow interests and highly repetitive behaviour [1]. Recently, unusual sensory processing has been included in the DSM-5 diagnostic criteria for ASC due to its high prevalence among those on the autism spectrum and its impact in their lives [2].

Anecdotally, individuals with high-functioning ASC often describe heightened sensory perceptions, across different modalities, which are experienced as over-arousing and overwhelming [3, 4]. Examples include accounts of people with ASC refusing to walk on grass because they find the smell overpowering [5], or who must wear the same clothes each day because any change in texture is sensed as uncomfortable [6]. The latter example may reflect tactile hyper-sensitivity or simply resistance to change. Others report they can hear the sound of electricity in the walls [7], or that they can read tiny text like the small print on the back of products from across a room [8]. In clinical studies, higher sensory reactivity and sensitivity scores have been shown in children with ASC compared to controls using the Dunn Sensory Profile [9] and in adults with ASC using the Sensory Perception Quotient [10]. Research with the Diagnostic Interview for Social and Communication disorders has reported that more than 90% of people with ASC showed sensory abnormalities, a rate much higher than controls [11]. Case studies in people with ASC have reported exceptional perception of absolute pitch in music and language [12, 13], and they are reported to have superior pitch sensitivity compared to controls [14]. Taken together, all these examples suggest that people with ASC sense the world in a different way compared with others.

Supporting this suggestion, there is limited experimental research suggesting enhanced sensory sensitivity in ASC across the modalities of touch [15, 16], vision [17–19], and audition [12, 14, 20]. For example, Blakemore et al. [15] carried out an experiment involving vibrotactile stimulation and found those with ASC had a lower perceptual threshold – i.e., tactile hypersensitivity – compared to controls. An experimental study of visual acuity reported by our lab [18] found that people with ASC were better than controls at perceiving the location of the gap in targets consisting of letter C’s presented on a computer screen, and has been replicated by a study from an independent lab [21].

However, there is a substantial gap in the literature on experimental research investigating olfactory detection thresholds in ASC. This is notable given all the anecdotal evidence mentioned above about sensory differences [3–13]. We are aware of only four experimental studies investigating olfactory processing in ASC which included detection threshold testing and used a comparison group [22–25]. While these studies reported no differences in olfactory detection sensitivity or impaired detection threshold for the ASC groups compared to controls, the main focus of two of them was on olfactory discrimination rather than detection sensitivity [22, 24]. Tasks involving the identification of odour choices involve different cognitive and neural processes compared to tasks examining ‘low-level’ olfactory detection thresholds registering presence or absence of an odour [26–28]. Three of the studies [23–25] involved a design with two or three different response options on each trial that the participants had to compare and remember. Since deficits in executive function are reported in ASC [29, 30], the inclusion of a number of choice options and the memory requirements during each trial may affect performance.

To fill this gap, the aim of the present study was to compare low-level olfactory sensitivity in adults with ASC to matched control participants using a standardised clinical measure of olfactory detection threshold where participants simply responded when the odour was barely detected. Based on previous findings of sensory hypersensitivity in other domains in ASC, we predicted people with ASC would show enhanced olfactory sensitivity compared to controls. We also investigated the relationship between olfactory hypersensitivity and degree of autistic traits in those with ASC, using scores from the Autism-Spectrum Quotient (AQ). Recent research has shown atypical sensory responsiveness is associated with greater social impairment in people with ASC [31–33]. Moreover, Hilton et al. [32] reported that olfaction was one of the strongest predictors of social impairment. As such, we predicted a correlation between olfactory sensitivity and AQ in the ASC group.

It is important to note that, in contrast to the various experimental studies mentioned above suggesting hypersensitivity in ASC across multiple sensory domains, other studies have failed to replicate such findings [34, 35]. There are also reports of sensory hyporesponsiveness in ASC [6] and, further, that hyporesponsiveness might actually better distinguish ASC from other developmental conditions [36]. In the tactile domain there is evidence that hyporesponsiveness shows a correlation with clinical symptoms that is not evident for tactile hyperresponsiveness [37]. A review of the sensory literature reported that hyporesponsivity might actually be more evident in ASC [38]. However, the research reviewed in that report [37] has been criticised for including physiological studies with very small samples [39]. It may also be the case that the behaviours of hyporesponsiveness may simply overlap with some of the social symptoms in ASC, such as a lack of response to being called by name. Therefore, further investigations about sensory processing in ASC using ‘low-level’ tasks with minimal cognitive and social demands are important to report in order to better understand how sensory processing might be different to controls.

## Methods

### Participants

We recruited 34 participants to take part in the study. The ASC group comprised 17 adult males (mean age =37.9 years, SD =13.4; mean Full Scale IQ =123.5, SD =10.8) who were recruited from the volunteer database of our centre (http://www.autismresearchcentre.com). They were all previously diagnosed with ASC (7 HFA/10 AS) according to international criteria [1] by qualified psychiatrists or clinical psychologists in recognized clinical centres. All participants with ASC further completed the AQ [40], a measure of autistic traits that has been found to be strongly predictive of a clinical diagnosis of AS according to DSM-IV criteria [41]. The AQ scores of the ASC group (mean AQ =38.9, SD =6.3, 94.1% scoring 32+) were similar to previously published studies (mean AQ score =35.8, SD =6.5, 80% scoring 32+) [40].

The control group comprised 17 adult males (mean age =27.2 years, SD =10.9; mean Full Scale IQ =122.7, SD =8.5) from the community. All participants in the study completed a measure of intelligence [42] and the resulting IQ scores did not differ between the groups: t(32) =0.25; *P* >0.05. However, the ASC group was significantly older than the control group: t(32) =2.54; *P* <0.02. Everyone who participated gave written informed consent to take part, and the study was approved by the Psychology Research Ethics Committee at Cambridge University. When asked, none of the participants reported having any problems affecting olfaction or any nasal congestion at the time of testing.

### Measures and procedures

Participants with ASC completed the AQ, a 50-item self-report questionnaire with a forced choice format asking about behaviours associated with autism. Each question includes the response choices ’Definitely agree’, ’Slightly agree’, ’Slightly disagree’, or ’Definitely disagree’. Approximately half the questions are worded to elicit an ’agree’ response from control individuals, and half to elicit a ’disagree’ response. The participant is scored one point for each question which is answered either slightly or definitely in a manner consistent with how a high-functioning person with AS would answer. An example question is ’I tend to have very strong interests which I get upset about if I can’t pursue’, which would score 1 if a participant chose either slightly agree or definitely agree. The range of scores is from 0to50, with higher scores indicating a greater degree of traits typical of ASC.

Participants also completed the Alcohol Sniff Test (AST), a standardised task developed at the UCSD Naval Dysfunction Clinic for measuring olfactory thresholds. The AST was used because it is a simple, rapid, and reliable evaluation of olfaction [43] and has been shown to have good test-retest reliability [44]. We followed the procedures as previously developed [43, 44], although in the present study we included a further three trials for each participant to get a better estimate of the parameter without overly increasing the time. We also included a greater range of detection distances because it was unknown beforehand what the mean detection distances would be for people with ASC, and our prediction was for larger mean detection distances for this group.

Commercially available antiseptic swabs of isopropyl alcohol (70% vol) were used as stimuli in the task. Alcohol swabs are well suited for the AST because at such concentrations and distances from the nose they do not exert trigeminal effects [44]. Activation of the trigeminal nerve could potentially confound results by providing an alternative sensory mechanism to the olfactory system. One swab was initially placed under the nose of participants to familiarise them with the alcohol odour. This was conducted in a separate area to where testing took place to ensure no odour remained in the area of testing. The location of testing had no air control mechanisms (e.g., air conditioning), which minimised air movement that might affect olfaction during the test. Testing was carried out ina lab space the furthestpossible from the location of any windows, and windows remained closed and covered throughout. A meter ruler was attached to the wall directly alongside where participants sat and in direct view for the experimenter. Participants sat in an adjustable chair, and the height of the chair was initially adjusted so that their nose was in line with the ‘zero’ mark on the ruler.

Participants were blindfolded and instructed to breathe normally throughout the task and were told when each trial was starting. At the beginning of each trial, the alcohol swab was placed in the central part of the participant’s abdomen in a vertical line directly below their nose. The alcohol pad was raised 1 cm vertically with each exhalation of the participant, based on visual observation of the chest rising and falling. The distance between the participant and the odour stimulus was varied by 1 cm distances until the odour was barely detected. Care was taken so the hand of the experimenter never came into contact with the participant, to ensure the participant was unaware about the location of the stimulus. A trial ended when the participant said “yes”, indicating they could smell the alcohol odour. Therefore, minimal involvement of executive function, memory, or other cognitive mechanisms was required during the task. The procedure was repeated five times per participant. The dependent measure was the mean distance (cm) from the nose measured when participants detected the smell. The mean distance was calculated by averaging the distances from the nose across all five trials for each participant. On one of the trials for each participant the experimenter delayed raising the stimulus for 5 seconds. This was included as a way of indexing the possibility that participants may have been making response biases in the clinical test, such as anticipatory errors or guessing. None of the participants reported detecting a smell during the ‘catch period’ of these ‘placebo’ trials, and the distance of eventual detection on these placebo trials did not differ significantly from the other trials (all *P* >0.05).

### Data analysis

A repeated-measures ANOVA was carried out on the mean olfactory detection distances to test the main hypothesis for differences in olfactory sensitivity, with Trial (1 vs. 2 vs. 3 vs. 4 vs. 5) as the within-subjects factor and Group (ASC vs. controls) as the between-subjects factor. As the sample in this study was relatively small, the data were re-analysed using a non-parametric bootstrapping procedure, to see if the effect was still present using a more conservative procedure. The ANOVA makes parametric assumptions about how the difference between the ASC and control groups would vary if this study were repeated. Specifically, it assumes that across many replications, the observed differences between two groups who are the same overall would follow a normal distribution. In contrast, bootstrapping procedures make no assumptions about how a statistic varies [45, 46]. Instead, an empirical estimate is obtained by repeatedly taking samples from the data that have been collected and seeing how the statistic of interest varies across these samples. In this case, the statistic of interest was the overall mean difference between the ASC and control groups’ scores, and the original pools of 17 ASC scores and 17 control scores were repeatedly sampled with replacement. This meant that a given person’s score could be included 0, 1, 2, or more times in each sample. This was repeated 10,000 times and on each occasion the mean difference between the ASC sample and the control sample was noted, providing many estimates of the group differences that might be seen in replications of this experiment.

## Results

**Figure 1:**
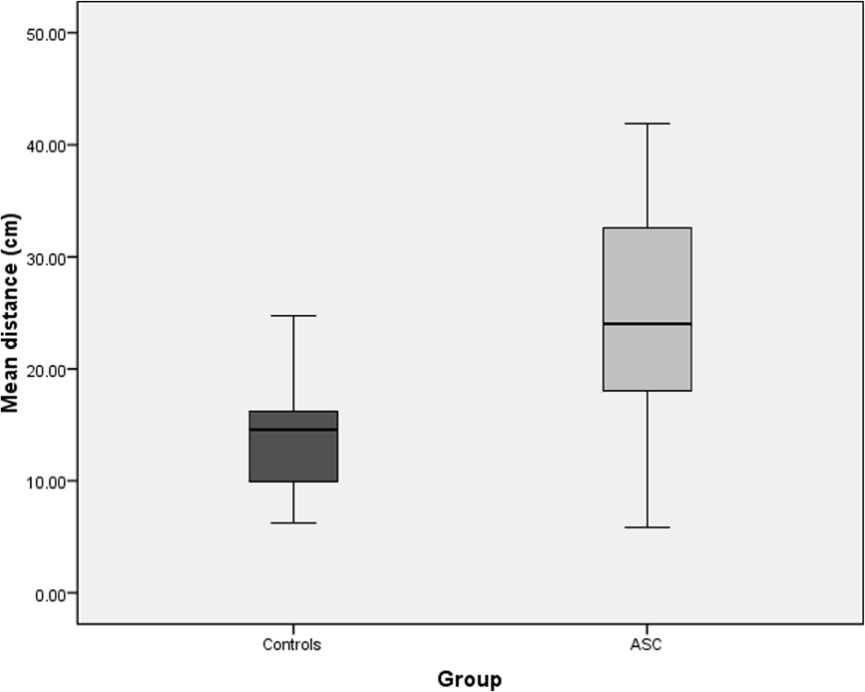
**Boxplot showing the mean distance (cm) from the nose at which the control and ASC groups were able to detect the odour in the Alcohol Sniff Test.** Larger values reflect greater perceptual sensitivity and error bars represent standard error values.

The Shapiro-Wilk test showed that both groups provided distributions of mean detection distance scores that were not significantly different from normal. Results of the repeated-measures ANOVA revealed the main effect of Trial was not significant, F(4,29) =0.32, *P* >0.05, η^2^_p_ =0.04, and that the interaction between Trial and Group was also not significant, F(4,29) =0.17, *P* >0.05, η^2^_p_ =0.02. Importantly, there was a significant main effect of Group, F(1,32) =10.1, *P* <0.01, η^2^_p_ =0.24, with the ASC group (mean =24.1 cm, SD =11.5, 95% CI =18.2 to 29.9) detecting the odour at a significantly further distance compared to the control group (mean =14.4 cm, SD =5.9, 95% CI =11.3 to 17.4; see Figure 1).

The bootstrap analysis confirmed that the mean advantage in detection distance for the ASC group was 9.70 cm and provided a 95% CI for this statistic of 2.90 to 15.93. This confidence interval was calculated using the simple percentile method, meaning the 10,000 recorded differences between ASC samples and control samples were arranged from the lowest to the highest and the cut-offs for the bottom 2.5% and top 2.5% of scores identified. In fact, of the 10,000 ‘replications’ in this analysis, in only 32 (0.32%) did the ASC group show no advantage over the control group, providing an empirical two-tailed estimate of *P* =0.0032 for the ASC group’s superior performance on this task.

**Figure 2:**
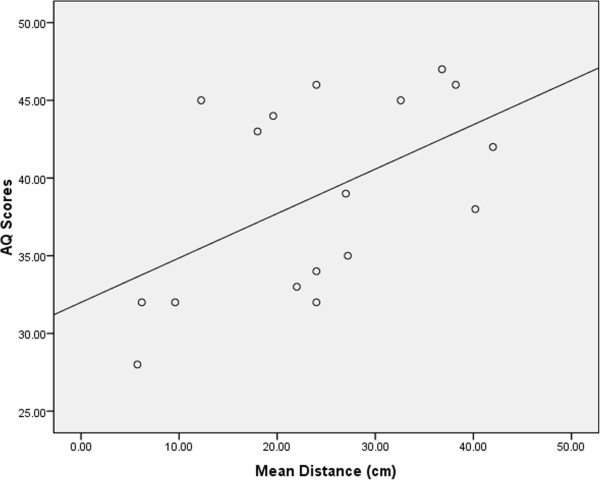
**Scatter plot showing the positive correlation between mean distance of odour detection thresholds (cm) and Autism-Spectrum Quotient scores (0 to 50) for the ASC group.**

Finally, correlation analyses revealed that mean odour detection distance was independent of IQ when the groups were combined (r =0.25, *P* >0.05) and when considered separately (ASC, r =0.32, *P* >0.05; controls, r =0.14, *P* >0.05). Similarly, there was no significant correlation between detection distance and age in either the ASC (r = -0.29, *P* >0.05) or control (r = -0.17, *P* >0.05) groups. However, there was a significant positive correlation in the ASC group between odour detection distance and AQ scores (r =0.52, *P* <0.05; Figure 2).

## Discussion

The present study provides the first experimental demonstration of enhanced olfactory sensitivity in people with ASC compared to controls. The ASC group detected the alcohol odour at a mean distance of 24.1 cm (SD =11.5), whilst controls were only able to detect the odour when it was 14.4 cm (SD =5.9) from the nose. The mean score by the control group is in line with previous results using the AST in samples of typical adults [43, 47] and the test showed a large effect size for the difference between the groups (Cohen’s *d* =1.06). The mean distance of detection in those with ASC was also positively correlated with AQ scores, showing that greater olfactory sensitivity was associated with a higher number of autistic traits. These findings extend previous experimental results of enhanced sensitivity across various modalities in people with ASC [14–20] to also include the olfactory domain. They also help interpret various anecdotal and questionnaire accounts of sensory hypersensitivity in ASC, which includes olfaction [3–10].

However, the present findings contradict previous experimental studies investigating olfactory thresholds in ASC, which have generally reported no differences compared to controls or deficits [22–25]. There are a number of factors that likely explain this. The main research focus of two of the earlier studies [22, 24] was on the discrimination of odours, not in determining thresholds for olfactory detection. One of the studies [22] did not include a task measuring olfactory threshold, and the other three studies [23–25] tested olfactory thresholds using a design that placed substantial memory requirements on participants. This is a concern sincepeople with ASC sometimes show deficits in executive function [29, 30]. As such, all these earlier studies used fundamentally different tasks than the present study. The AST assessed only ‘low-level’ olfactory detection thresholds with minimal cognitive task demands, and as such is a ‘purer’ test if the focus of interest is on detection ability.

Previous olfaction detection studies in ASC also used 1-butanol for the odour in their methods, which is known to stimulate the trigeminal nerve at the concentration levels and distances involved [44]. This means results could have been affected by activation in alternative sensory pathways to the olfactory system. The participant groups in the Suzuki study were able to detect the odour within the first 4 out of the 10 concentration increments included, suggesting the odour concentrations may have been too easy to detect. Participants in some of the previous studies of olfaction in ASC [22, 23] were also generally younger and had lower levels of cognitive functioning than participants in the current study. Differences in the detection methods and samples used between those studies and the present research makes it hard to directly compare results and may help explain inconsistent results in the literature. Given the methodological issues just described, the results presented here may be more reliable as measures of detection threshold.

The present study further showed a quantitative relationship between level of enhanced olfactory sensitivity and the degree of autistic traits. Amongst participants with a clinical diagnosis of ASC, olfactory sensitivity was higher in those with more severe ASC traits. Moreover, no such relationship was seen for either age or level of cognitive functioning. This is consistent with results from questionnaire-based research looking at sensory processing across different modalities, which have reported relationships between atypical sensory responsiveness and degree of social impairment [31, 32]. The present results extend the previous research findings showing a relationship between sensory dysfunction and ASC traits to include olfaction. However, the findings in the present study contradict those of an earlier experimental study that looked specifically at olfactory sensitivity and its relation to autism severity [48]. The authors utilised data from their own previous research on olfaction processing which reported no group differences in olfactory detection threshold [23], and further reported no relationship was evident between olfactory detection thresholds and autism severity [48]. An explanation for the difference in results with the current study might be of the use of research involving children and adolescents across different developmental stages [23, 48], whereas the present study included adults. Given that some have suggested that the olfactory system develops differently in ASC compared to controls [49, 50], this difference in age may explain the discrepancy of the results.

The present study included a large variability in scores across the ASC group, and the scores overlapped with those of the control group. While research has shown that olfactory sensitivity is quite common and is evident in over half of people with ASC [51], it also indicates that differences in olfactory functioning are not apparent in all people with ASC. This variability suggests other factors could interact with the olfactory system and play a role in the degree of sensory sensitivity in ASC. One potential factor that could be important for olfactory processing is the perceived pleasantness of the odour stimuli for people with ASC [23]. Research has found that the degree of pleasantness rated for different odours in ASC was related to how easily they identified odours, with more pleasantly-rated odours being easier to identify, and more unpleasant odours identified with greater difficulty [52]. Therefore, it is possible the pleasantness level of odours for people with ASC could also affect their detection threshold for that odour. The participants in the present study did not rate the pleasantness of the odour stimuli used in the task, so its role in the results cannot be determined. People with ASC also show differences in neural habituation to repeated stimuli, generally showing reduced habituation compared to controls [53, 54]. There is also evidence that reduced habituation in ASC affects sensory processing and may play a role in atypical sensory sensitivity [55]. However, the present findings within olfaction do not support the idea that differences in habituation were involved in the results, as there was no evidence for differences in habituation or learning effects across the trials for those with ASC.

An important question remains about how enhanced low-level processing of sensory information might contribute to characteristic deficits in social and communication functioning in ASC, if indeed they do, rather than arising as an epiphenomenon. One idea is that enhanced low-level sensory processing may result in a flood of hyper-detailed information from the environment [56], interfering with social processing. A large amount of research has shown superior attention to detail in ASC, such as better performance compared to controls on the Embedded Figures Test [57]. We suggest that enhanced sensory processing is associated with the greater attention to detail and processing of features that are characteristic of ASC. This cognitive style may be beneficial for some tasks, such as visual search paradigms. However, it may impair performance on other tasks, like those involving mentalizing, especially when there are no details with which to draw inferences. Visual search benefits from attention to detail because there are real details to use in order to verify the facts. Mentalizing does not benefit from attention to detail because mental states cannot be directly observed and can only be inferred from contextual cues (e.g. facial expression, vocal intonation, recent events, and perceptual access). This means there are few, if any, facts that can be verified during mentalizing.

Another possibility is that over-selective or over-focused attention could lead to sensory over-reactivity, with enhanced processing of sensory stimuli occurring within an amplified focus of attention towards sensory information [39, 58]. This suggests that one mechanism (e.g. attention) could potentially produce sensory-processing differences across multiple modalities within ASC, and that other factors contribute to the heterogeneous profiles typically seen [59]. A recent study looked at self-reports of sensory processing in a large sample of 221 adults with ASC and reported sensory over-responsivity across all major sensory systems, and found that the sensory over-responsivity was related to degree of autism traits across each modality [60]. These findings are consistent with the idea of one common mechanism contributing to differences in sensory sensitivity across all modalities, as it is unlikely that separate mechanisms within each of the major sensory systems would independently show the same type of dysfunction. However, further research replicating these findings using psychophysiological measures would help validate this idea.

In terms of neurophysiology, a brain region hypothesised to be involved in sensory over-reactivity and hyperattention in ASC is the amygdala [39]. This is relevant given the theories and research linking dysfunction of the amygdala to the social-emotional difficulties in ASC [61–64]. The amygdala is also important for olfaction processing, as it receives information directly from the olfactory bulb. Up to 40% of amygdala neurons in rat brains show activity in response to olfactory stimuli [65]. Similarly, electrophysiological studies in humans have also revealed that amygdala neurons are responsive to odours [66, 67], and that activity of the amygdala is shown to produce auras in the olfactory domain [68, 69]. Temporal lobectomy that includes the amygdala region is associated with deficits in processing odours [70, 71]. Therefore, in addition to the amygdala’s association with deficits of social-emotional processing in ASC, atypical amygdala functioning might also affect the processing of olfactory information.

Certain neuroendocrine and neurotransmitter systems may also play an important role in enhanced olfactory acuity in ASC. Studies with animals have shown the olfactory bulb has a high concentration of estrogen and androgen receptors [72], and receptors for these neurohormones are found in cells known to facilitate odour discrimination [73, 74]. In rats, testosterone plays a significant role in enhancing olfactory acuity [75], and reduced testosterone production (e.g. via castration) results in decreased olfactory sensitivity [76]. It has been proposed that higher levels of prenatal androgens may be a causal factor in ASC [77–80]. There is also much evidence for impairments in γ-aminobutyric acid (GABA) neurotransmission in ASC [81, 82]. GABA is the main inhibitory neurotransmitter in the human brain and reduced GABA functioning could produce imbalances in the excitation and inhibition in sensory systems leading to enhanced sensitivity. Therefore, altered GABA functioning could potentially help explain the current findings.

It could be argued that, rather than reflecting differences in olfactory sensitivity between the ASC and control groups, the results here are an experimental artefact. Given the design of the AST, it is possible the group difference in olfactory sensitivity may have emerged from a response bias by the ASC group. However, to counter such a claim we included a catch (‘placebo’) trial for every participant in the study in order to test for response biases, where the raising of the stimulus towards the nose was delayed by 5 seconds. Not once did any participant with ASC respond during this catch period, suggesting response biases where people with ASC may have been simply guessing about the presence of the stimulus were not present. In addition, the eventual detection distances for the catch trials did not differ from other trials where no catch period was present. These findings with the catch trials suggest no response bias existed for those with ASC, where they may have responded in expectation of the odour stimulus. Moreover, to reduce the chance of a type I error from the relatively small number of participants (n = 17 for each group) and trials, the data were further analysed using a non-parametric bootstrapping procedure. The bootstrapping technique is appropriate for datasets with fewer participants and trials, which involved looking at the mean difference between groups in 10,000 samples of the data. Results showed that 99.68% of the time a greater detection distance was found for the ASC group, suggesting the present results are unlikely to represent a type I error.

Some limitations of the present study should be noted. First is the relatively small numberof participants (n =34), which included 17 people with ASC and 17 controls. Although this number is in line with many previously published studies testing sensory sensitivity in ASC, and although we conducted an additional analysis to compensate for the small sample, further research should include larger samples. While the present study involved a standardised clinical test of olfaction sensitivity, this test involved fewer trials than other methods for testing sensory thresholds. Further research on olfaction in ASC should utilise other methods of olfactory threshold detection to replicate the current findings, as well as alternative designs such as staircase methods and signal detection theory [83]. The two groups did differ between each other in terms of mean age, although this was not considered a problem since our prediction was for superior olfaction in the ASC group and olfactory sensitivity declines with age [84, 85]. Therefore, if the ASC group showed hypersensitivity in olfaction despite being older, this would constitute a strong, conservative test of the hypothesis.

Another limitation is that alcohol was the only olfactory stimulus used in the present task, which limits making generalisations about the results beyond this odour. This is particularly relevant given the earlier discussion of how people with ASC might be sensitive to odour pleasantness. Additional research including other types of olfactory stimuli is needed. Further experimental research should also be run alongside self-report measures of sensory processing in the same individuals, as this would allow the experimental data to be correlated with self-reports about sensitivity. The AQ was not included for the control sample, so we cannot rule out that some of them may have had higher traits of ASC. Further research should include formal clinical confirmations of diagnosis for both groups using theAutism Diagnostic Observation Scheduleand Autism Diagnostic Interview-Revised, which was a limitation to the current research. This would help verify clinical diagnosis for the ASC group. A final limitation is that the research here focused only on participants who were high-functioning adult males, diagnosed with either AS or HFA. Since the AST is a simple and reliable measure of olfactory detection, this task could be used to determine olfaction thresholds across other autism spectrum groups including females, those of younger ages, and individuals with lower-functioning ASC.

## Conclusions

The present results show enhanced olfactory sensitivity in ASC, with the degree of olfactory hypersensitivity being associated with the severity of ASC traits. These findings within olfaction are consistent with reports of enhanced sensitivity in other modalities, as well as clinical reports of enhanced sensations that include olfactory processing. The underlying neurophysiology could involve the amygdala and certain neuroendocrine and neurotransmitter systems, including testosterone and GABA. These are reported to be involved in both olfaction and ASC.

## Abbreviations

AQ: Autism-Spectrum Quotient
AS: Asperger syndrome
ASC: Autism spectrum conditions
AST: alcohol sniff test
GABA: γ-aminobutyric acid HFA, High-functioning autism.
